# Classification of hand and wrist movements via surface electromyogram using the random convolutional kernels transform

**DOI:** 10.1038/s41598-024-54677-7

**Published:** 2024-02-19

**Authors:** Daniel Ovadia, Alex Segal, Neta Rabin

**Affiliations:** 1 Department of Biomedical Engineering, Tel Aviv University, Tel Aviv, Israel; 2 Afeka Tel Aviv Academic College of Engineering, Tel Aviv, Israel; 3 Department of Industrial Engineering, Tel Aviv University, Tel Aviv, Israel

**Keywords:** Biomedical engineering, Data processing, Biomedical engineering, Data processing

## Abstract

Prosthetic devices are vital for enhancing personal autonomy and the quality of life for amputees. However, the rejection rate for electric upper-limb prostheses remains high at around 30%, often due to issues like functionality, control, reliability, and cost. Thus, developing reliable, robust, and cost-effective human-machine interfaces is crucial for user acceptance. Machine learning algorithms using Surface Electromyography (sEMG) signal classification hold promise for natural prosthetic control. This study aims to enhance hand and wrist movement classification using sEMG signals, treated as time series data. A novel approach is employed, combining a variation of the Random Convolutional Kernel Transform (ROCKET) for feature extraction with a cross-validation ridge classifier. Traditionally, achieving high accuracy in time series classification required complex, computationally intensive methods. However, recent advances show that simple linear classifiers combined with ROCKET can achieve state-of-the-art accuracy with reduced computational complexity. The algorithm was tested on the UCI sEMG hand movement dataset, as well as on the Ninapro DB5 and DB7 datasets. We demonstrate how the proposed approach delivers high discrimination accuracy with minimal parameter tuning requirements, offering a promising solution to improve prosthetic control and user satisfaction.

## Introduction

Surface electromyography (sEMG) is a non-invasive technique for measuring the electrical activity of muscle groups on the skin surface. The usage of sEMG for clinical diagnostics began in the 1960s. Nowadays, sEMG plays a central role in many applications, including clinical diagnostics, human-machine interactions and more. One such realm where sEMG plays a crucial role is control for prosthetic devices. Prosthetic devices play one of the key factors in personal autonomy, by affecting the life quality for amputees. During the past decade, multiple prosthetic hands have become available in the market, yet the rejection rate for electric upper-limb prostheses is roughly 30% due to dissatisfaction about issues like function, ease of control, reliability, and cost^1^. Therefore, achieving a high level of reliability, robustness, and low cost of human-machine interfaces is important for user experience and their acceptance of the prosthetic hand. A machine learning algorithm based on sEMG classification could potentially allow the user a natural control of the prosthesis.

Various control methods for prosthetic hands have been introduced and investigated. Among them are sEMG, as mentioned above^2–7^, electroneurography (electroneurographic signals, requiring an interface directly with the peripheral nervous system or the central nervous system)^8–10^, mechanomyography (measures the vibrations of muscle fibres during motion)^11–13^, and force myography, which detect changes in the pressure patterns between the limb and socket caused by the contraction of the forearm muscles)^14–17^. Two major advantages of sEMG are existence of low-cost options, and its non-invasive nature.

Our objective is to improve the classification of the hand and wrist movements based on the sEMG signals, represented as time series. To this end, we apply a novel approach, using a variation of Random Convolutional Kernel Transform (ROCKET)^18,19^ as a feature extraction method, combined with a cross-validation ridge classifier. The majority of the methods for time series classification which achieve state-of-the-art accuracy have high computational complexity and extensive training time even for smaller data sets. With the recent success of convolutional neural networks for time series classification, it also been shown that simple linear classifiers using ROCKET can achieve state-of-the-art accuracy with much less computational expense (see Ref.^18^). In order to further improve the classification, we suggest to couple the ROCKET features with a cosine similarity matrix that codes the relationship between the measured channels. This matrix is computed based on the ROCKET representation, and it is a complementing way to code the movemoent pattern.

The performance was evaluated on three different datasets. The first dataset, taken from the UCI repository^20^, was previously used by Sapsanis et al.^21^. The data contains 6 movements recorded by two channels that measured the electrical activity of the hand muscles at 500 Hz. The second dataset is the NinaPro DB5 and DB7 data sets. DB5 includes 53 movements using 16 electrodes (signal channels)^4^. DB7 includes a subset of DB5’s movements. It uses 12 electrodes, and was tested on 22 participants, 2 of them are amputees. The proposed model achieved accuracy of $98\pm 2.54\%$ for the UCI dataset. For the NinaPro DB5 dataset, accuracies of $93.65\pm 2.99\%$ for the 8 channels data and $98.27\pm 1.35\%$ for 16 channels data were achieved. Accuracies of $97.97 \pm 3.88\%$ were achieved for the DB7 dataset, however, the avarage accuracy for the amputee subjects was $87\%.$

The results were compared with the ones reported in Ref.^22^, and with feature based calssifiers, taking features that were suggested in Refs.^23^ and ^24^. We show that the suggested ROCKET based algorithm outperform previous studies’ results while classifying a larger amount of hand and wrist movements. To the best of our knowledge, this is the first time that ROCKET / miniROCKET is being utilized for classification of sEMG signals.

The rest of this paper is organized as follows. Section "Related work" overviews recent related work. In Sect. "Methods" the utilised methods and dataset are described. The proposed algorithm is given in Sect. "ROCKET based classification of sEMG hand and wrist movements". Section  Experimental results details the experimental results. Last, conclusion are discussed in Sect. Conclusion.

## Related work

This work continues multiple previous attempts to implement a movement classification algorithm using sEMG as our main input. Attempts to control hand prostheses were made already in the late 60s^25^. Since then, many new approaches and developments were proposed. These methods may be coarsely branched into two categories, depending on the way features are computed. The first is based on feature computing and engineering, constructing features that capture the characteristics of the signal in the time and frequency domain. Then, machine learning or deep learning tools are applied on the feature space for classification. The second is deep learning approaches, which take the signal as input and build one unified machine to learn features and classify the signals based on the formed representation. In what follows, recent papers that follow either the feature engineering or feature learning approach are reviewed. In addition, we point out to recent papers that incorporate ideas from the deep learning models into feature extraction based techniques.

The work of Phinyomark et al.^26^ examined 37 time and frequency domain features for EMG signal classification tasks and idetified strong and redundant features. They grouped the features into four categories based on what the features capture, these are, energy and complexity information methods, frequency information method, prediction model method, and time-dependence method. Features within each group are redundant. In general, a small number of time domain features were shown to perform better than using a small number of frequency domain features. Mean absolute value (MAV), waveform length (WL), and Willison amplitude (WAMP) from the first and secound group, where shown to produce robust and accurate classification results. In a later paper, Phinyomark et al.^27^ examined the classification accuracy of myoelectric pattern recognition when the train and test samples are recorded over a relatively long time period. To do so, 50 time and frequency domain features were computed. Sample entropy was found to be a strong and stable feature, additional robust features are the cepstral coefficients (CC), modified mean absolute value, root mean square (RMS) and WL. Some of these were proposed and investigated in earlier work^26,28,29^. A further analysis of the feature space that is commonly used for EMG classification was carried out in Ref.^30^. Topological tools were used to create charts from 58 features over several datasets. The method selects representetive, non-redumdant features. Some of these identified features are WL, difference absolute mean value (DMAV) and difference absolute standard deviation value (DASDV) from the energy features group, maximum fractal length (MFL), sample entropy and WAMP from the non-linear and frequency group.

The work of Pizzolato et al.^4^ aimed to compare between six different sEMG setups using identical hand movements classification task. Features were extracted from overlapping windows. The following five features, which were also used in previous work^31,32^, were computed. These are RMS, time domain statistics^33^, Histogram, Marginal discrete wavelet transform and the concatenation of all these features. Then, machine learning algorithms, such as support vector machine (SVM) and random forest, were applied for classification (accuracy of 69.04%). In Ref.^23^, the authors consider a set of features that perform well in taking into account force level variables. This type of analysis is important for transradial amputees. A set of features that are invariant to the force level are identified, there are modified spectral moments, which are based on features that were suggested in Ref.^34^.

Jiang et al.^35^ preformed optimization for classification of high-density sEMG signals (256 channels) by feature extraction and data augmentation methods. 50 known temporal-spectral-spatial domain features, including a new introduced feature denoted by the spatial synchronization (SS) feature, which measures the synchronization of waveforms between neighbor channels were used. 15 feature optimization techniques, and seven classifiers were evaluated for classification of 35 hand gestures. The SVM classifier achieved the highest classification accuracy (91.9%), this result was achieved with an optimal feature set that includes the SS feature along with ten other features.

Sri-iesaranusorn et al.^22^ applied a deep neural network to a set of computed features that were sectioned using a sliding window. Features included RMS, MAV, WL, mean absolute value slope, zero crossings and slope sign changes. The model was evaluated on a publicly available database from the Ninapro project (sEMG databases for advanced hand myoelectric prosthetics). The data set included sEMG signals for 41 different hand movements (from Ninapro DB5 and DB7^4^). The classification accuracy was $93.87\% \pm 1.49\%$.

Time frequency features, like the short time Fourier transform (STFT) and wavelet based representations were investigated in several papers. In Rabin et al.^36^, the feature space was composed of STFT matrices of the signals. The data set included sEMG signals from 5 subjects who perform 6 different hand movements. The dimension of the STFT was reduced with principal component analysis (PCA) and diffusion maps. K-nearest neighbors (KNN) was used for classification ($94.8\% \pm 3\%$). A different approach by Shi et al.^37^ suggested a feature extraction method based on the wavelet packet transform (WPT) and principal component analysis (PCA) for reducing the dimension of the feature vector. The results were compared with a model that uses computed features: MAV, RMS and the wavelet transform coefficient (WTC). Additionally, the authors proposed a method based on the scale unscented Kalman filter (SUKF) and neural network (NN) that was used for lower limb motion classification (average accuracy - 93.7%). In a recent paper^38^, the deep wavelet scattering transform (WST) was applied for EMG pattern recognition. The main advantage of WST is its invariant properties, which makes this transform robust in terms of distortions. The results were shown to outperform features that were extracted by the wavelet transform (WT) and the wavelet packet transform (WPT).

Deep learning approaches create features from the input signals, these features are optimized to solve the defined learning task. Several papers utilize an image-type input. For example, in Ref.^39^, a convolutional neural network was applied on spectrograms calculated from the signals. Results were evaluated on the NinaPro DB2 and DB3 datasets, and showed to achieve improved accuracy compared to SVM based classifiers. While CNN models manage to capture the saptial relationship of the input, they don’t have the ability to code long term temporal relationships. Long Short-Term Memory (LSTM) can overcome this limitation. Karman et al.^40^ proposed a hybrid CNN and LSTM model for classification of hand activity. Multi-channel EMG signals were first fed into convolutional layers, followed by bi-directional LSTM layers. Then, two fully connected layers were evoked for classification. Experimental results were reported for several NinaPro datasets and the UCI gesture dataset and classification accurcy was shown to improve the state of the art methods. Another recent paper that aims to capture both spatial and spectral dependencies in by Shen et al.^41^. The authors applied a Convolutional Vision Transformer and Stacking Ensemble Learning for sEMG hand movements classification. This method allows fusion of sequential and spatial features of sEMG signals with the parallel training. The evaluated data sets were from the Ninapro database (49 movements from DB2 and 12/17 movements from DB5, 80.02% and 76.83%/73.23%, respectively). Last, in Ref.^42^ a spatio-temporal framework extended the well known Dynamic time wrapping (DTW) similarity to operate in a spatial setting and was combined with an LSTM model. Results were provided for four public datasets, including some from the NinaPro project, and were shown to be more accurate than other deep learning methods. However, since DTW is computationally expensive, the method may not be the most suitable for real time classification.

Deep learning models have shown advantages in learning both spatial and long term temporal connections. Nevertheless, their black box nature, together with the need for a large training set and computational complexity, remain a limitation. To overcome this drawback, the work of Khushaba et al.^24^ adapts ideas from the deep learning framework and combines them with feature extraction techniques. Feature extraction was carried out as a first step, where the features were stored in a matrix representation. These feature matrices went under spatio-temporal convolutions that enabled to learn short and long term temporal dynamics. The method was tested on the DB5 and DB7 NinaPro datasets, it benefits of low computational cost and was shown to improve the accuracy of deep learning models.

Following the spirit of Ref.^24^, seeking for robust time series classification techniques that enjoy a low-computational cost, we mention several new methods that may be suitable for coding EMG signals. Among them, Shapelets based algorithms^43^, Random Convolutional Kernel Transform (ROCKET) with its variations^18,19^, combinations of Markov Transition Field (MTF), Gramian Angular Field (GAF) and various neural networks^44–46^. Shapelets are a family of algorithms that focus on finding short patterns, called shapelets, appearing anywhere in the time series. A class is then distinguished by the presence or absence of one or more shapelets somewhere in the series^47^. The MTF and GAF encode times series to 2D images, which can serve as an input to a neural network^46,48^. ROCKET algorithms use a large number of random convolution kernels in conjunction with a linear classifier (ridge regression or logistic regression). Every kernel is applied to each instance. From the resulting feature maps, the maximum value and a novel feature, proportion of positive values (PPV), are returned (see details in Sect. "Methods"). A study by Ruiz et al.^49^ compared multiple times series classification algorithms on various data sets and concluded that ROCKET is the recommended choice for time series classification due to high overall accuracy and remarkably fast training time. As stated above, in this work we evaluate the performance of the ROCKET transform as a new way for feature extraction for sEMG signals. The transform computes many features, this resembles the type of information that is learned by a CNN. However, since there is no network to train, the computation is fast and simple and also fits datasets of limited size. In addition, in this work we followed a setting in which the ROCKET features are extracted from the entire movement (without using overlapping windows), thus, the features capture long range temporal information. Additionally, multi-channel relationships were added into the model as additional features.

## Methods

This section provides the essential mathematical background for the ROCKET and channel similarity techniques, which were utilized in this work, as well as the dataset.

### ROCKET and MiniROCKET

At its core, ROCKET is a a method for time series transformation, or feature extraction^18^. The extracted features contain information related to series class membership, which can be modeled by a linear classifier. By default, ROCKET transforms time series by applying convolution with 10, 000 random convolution kernels which have random length chosen from $\{7, 9, 11\}$, weights drawn from the standard normal distribution ${\mathcal {N}}(0,1)$, bias drawn uniformly from ${\mathcal {U}}(-1,1)$, dilation and padding chosen randomly. For feature extraction, ROCKET uses using global max pooling and proportion of positive values (PPV).

Denote by *X*(*t*) the time series vector of length *n*, by $\omega$ the convolution kernel and the bias vector by *b*. Then, the global max pooling is defined by

1 $$\begin{aligned} GMP = \max _{i} (X *\omega )(i) \end{aligned}$$

where

2 $$\begin{aligned} (X *\omega )(i) = \sum _{j=0}^{l_{kernel}-1} X(i+j)\cdot \omega (j) \end{aligned}$$

and

3 $$\begin{aligned} PPV = \frac{1}{n} \sum _{i}\left[ X*\omega - b > 0\right] \end{aligned}$$

This way, each kernel generates two features for a given time series *X*(*t*),  resulting in approximately 20, 000 features per time series.

miniROCKET (MINImally RandOm Convolutional KErnel Transform)^19^ is a nearly deterministic reformulation of ROCKET that is roughly 75 times faster on larger datasets and with roughly equivalent accuracy. Recently it has become the default implementation of ROCKET. MiniROCKET uses only kernels of length 9 with weights drawn from the set $\{-1,2\}$ so that their sum is 0. This implies a total of 84 possible kernels, before dilation and bias. Limiting the structure of the kernels allows a significantly faster computation. The exact value of the two selected kernel weights $\{-1,2\}$ is not important as long as the kernel weights sum to 0, which ensures that the kernels are sensitive only to the relative magnitude of the input. For each convolutional kernel, the bias is drawn from the convolutional output of one random training sample. Note that the bias selection is the only random component of the MiniROCKET. In addition, MiniROCKET uses only the PPV feature (see Eq. (3)) and omits the global max pooling (see Eq. (2)). Reducing the pooling step to a single feature yields approximately 10, 000 features per time series.

The computational complexity of ROCKET and MiniROCKET is linear in the number of kernels, the number of training examples and the time series length, formally $O(\text{ num. } \text{ of } \text{ kernels } \times \text{ num. } \text{ of } \text{ samples } \times \text{ signal } \text{ length})$. In terms of space complexity, ROCKET doesn’t store any intermediate values. MiniROCKET stores 13 additional copies of each input time series signal.

### Cosine similarity

Cosine Similarity is a measure of similarity commonly used in data analysis for comparison of two finite series. To this end, the sequences *X*(*n*), *Y*(*n*) are represented as vectors in ${\mathbb {R}}^d$, where *d* is their length. Then, the cosine similarity of *X* and *Y* is defined by

4 $$\begin{aligned} S_C(X,Y) = \frac{\langle X, Y \rangle }{\Vert X\Vert \cdot \Vert Y\Vert }. \end{aligned}$$

Note that $S_C(X,Y)$ is cosine of the angle between *X* and *Y*, and thus it results in similarity range from $-1$ (exactly opposite) to 1 (the same).

### Ridge regression and classification

Ridge regression is a modification of regular linear regression, which enables reducing the influence of less important features, using $L_2$ regularization (see Ref.^50^). Assume we have a linear model $Y = \sum _{i=1}^{n} b_i X_i.$

Using ordinary least squares, we can find $(b_i)$ by minimizing $\arg \min _B \Vert Y - XB \Vert _2^2,$ where *B* is the vector $(b_1, \ldots b_n)$. The key difference for Ridge regression is using an $L_2$ penalty term, and minimizing the following term

5 $$\begin{aligned} \arg \min _B \Vert Y-XB \Vert ^2_2 + \lambda \Vert B\Vert _2^2. \end{aligned}$$

Notice that case $\lambda = 0$ is the ordinary least squares. On the other hand when $\lambda$ tends to $\infty$, the coefficients tend to 0, which will imply under-fitting. Thus, choosing the correct $\lambda$ is a key factor in classification.

The above provides an algorithm for classification (without over-fitting) in cases where there are significantly more features than samples. To this end, a set of possible values for $\lambda$ was chosen. For each value, Ridge regression was preformed, leaving one of the samples out for validation. This is known as Cross Validation Ridge Classifier. Leave-one-out cross-validation is a special case of cross-validation where the number of folds equals the number of instances in the data set. Thus, the learning algorithm is applied once for each instance, using all other instances as a training set and using the selected instance as a single-item test set. We use a ridge regression classifier, which has the advantage of fast cross-validation for the regularization hyper-parameter and works very well when the data-set is not very large compared to the number of features.

### The dataset

**Figure 1:**
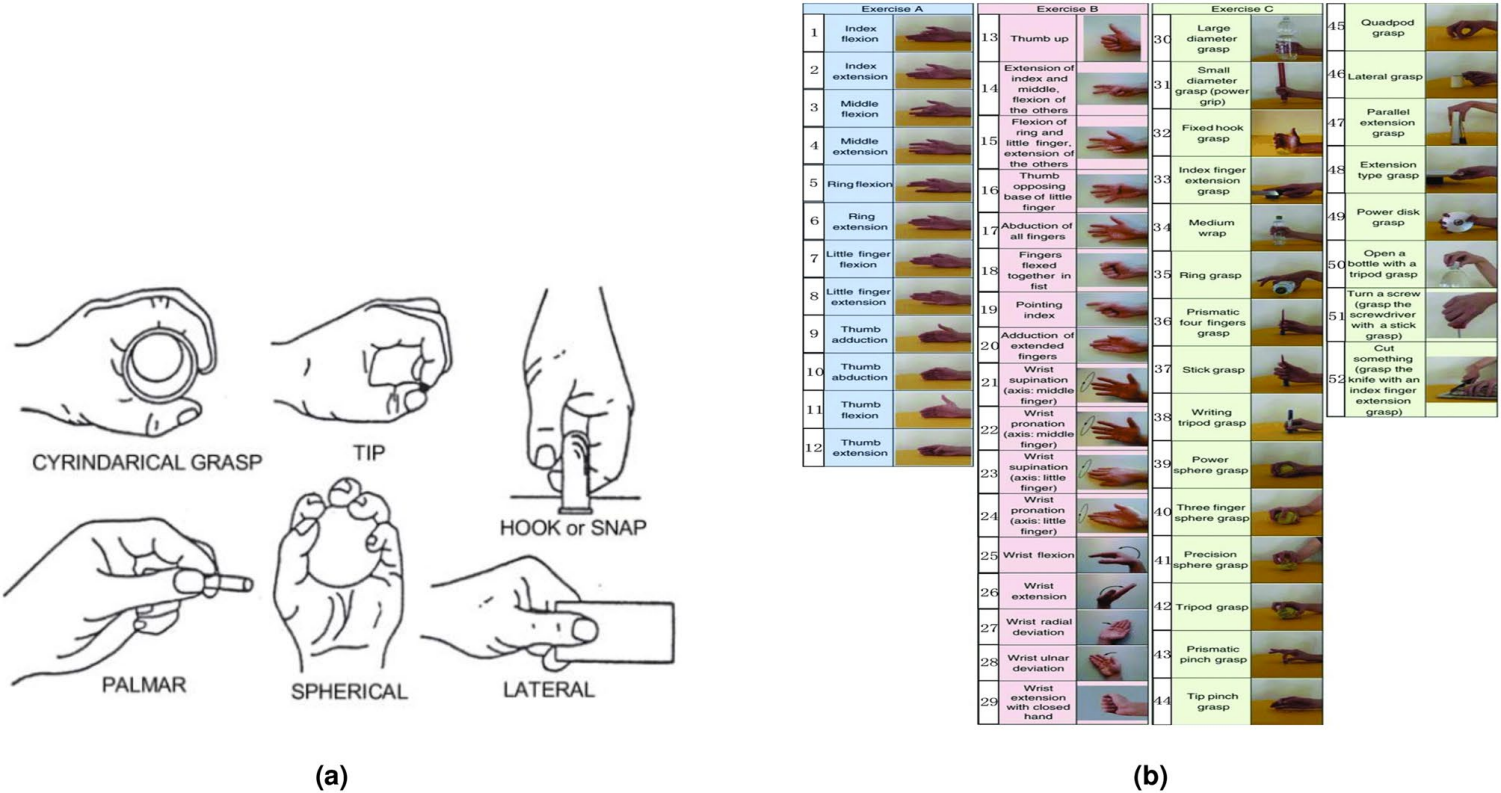
Dataset movement types. (**a**) Six hand movements, UCI dataset^20^. Licensed under a Creative Commons Attribution 4.0 International License. (**b**) Hand gestures of the NinaPro database. Licensed under a Creative Commons Attribution 4.0 International License.

The first dataset, taken from the UCI repository, was obtained by taking sEMG measurements from 5 subjects while performing the following 6 movements (see Fig. 1a):

- Holding cylindrical tools (Cylindrical).
- Supporting a heavy load (Hook).
- Holding small tools (Tip)
- Grasping with palm facing the object (Palmar).
- Holding spherical tools (Spherical) and f) holding thin objects (Lateral)

Each movement was recorded by two channels that measured the electrical activity of the hand muscles at 500 Hz. Subjects were asked to repeat each movement for 30 times. The recordings included only the records of the muscle activity, meaning there were no need for segmentation.

The second data set used in the work was taken from the Ninapro database. It’s a publicly available multi-modal database to foster research on human, robotic and prosthetic hands and on machine learning based control systems. We focused on the DB5 dataset but show some results on the DB7 datasets, which includes two amputee subjects. DB5 was recorded with two Thalmic Myo armbands (see Ref.^4^). Each Myo armband has 8 sEMG single differential electrodes (a total of 16, however, the database can be used to test the Myo armbands separately as well). The top Myo armband is placed closed to the elbow with the first sensor placed on the radio humeral joint, as in the standard Ninapro configuration for the equally spaced electrodes; the second Myo armband is placed just after the first, nearer to the hand, tilted of 22.5 degrees. This configuration provides an extended uniform muscle mapping at an extremely affordable cost. During the acquisition, the subjects were asked to repeat the movements with the right hand. Each movement repetition lasted 5 seconds and was followed by 3 seconds of rest. The protocol includes 6 repetitions of 52 different movements performed by 10 intact subjects. The sampling rate was 200 Hz. The movements were selected from the hand taxonomy as well as from hand robotics literature (see Fig. 1b). For DB7, there are 12 sEMG input channels of Delsys Trigno electrodes. Like in DB5, there were 6 repetitions. The movemebts are a subset of those described in DB5, and the sampling rate is 2 kHz.

## ROCKET based classification of sEMG hand and wrist movements

Each repetition of each movement was divided from the full signals according to the re-stimulus indices, which is the corrected stimulus, processed with movement detection algorithms^4^. Due to the subject’s response time, there is a variability in the movement’s time interval. The purpose of this process is to extract signals representing movement only. For each subject, the longest repetition between all movements was found. According to this maximum length, zero padding was applied to the rest of the signals.

Our first classification step was separating between movements and rest periods. The separation between movement and rest periods resulted in high accuracy rate (roughly 99%, see Table 1). Thus, it was decided to eliminate the rest data from the final movement classification. This results in a more reliable accuracy rate of movement classification. For the next processing steps, the MiniROCKET transform is applied to entire detected movement, bypassing the need to separate the signal into overlapping windows, and, considering the long-term dynamics of the movement.

**Table 1:** MiniROCKET classification - rest vs. movement.

#	Channels, rand. split	Weights	Validation accuracy	Test accuracy
1	16, Y	(1, 0)	$98.58\pm 0.72\%$	$98.49\pm 0.88\%$
2	16, N	(1, 0)	$98.54\pm 0.64\%$	$98.77\pm 0.74\%$
3	16, Y	(0.3, 0.7)	$98.74\pm 0.74\%$	$98.6\pm 0.89\%$
4	16, N	(0.3, 0.7)	$98.67\pm 0.65\%$	$98.96\pm 0.67\%$

**Figure 2:**
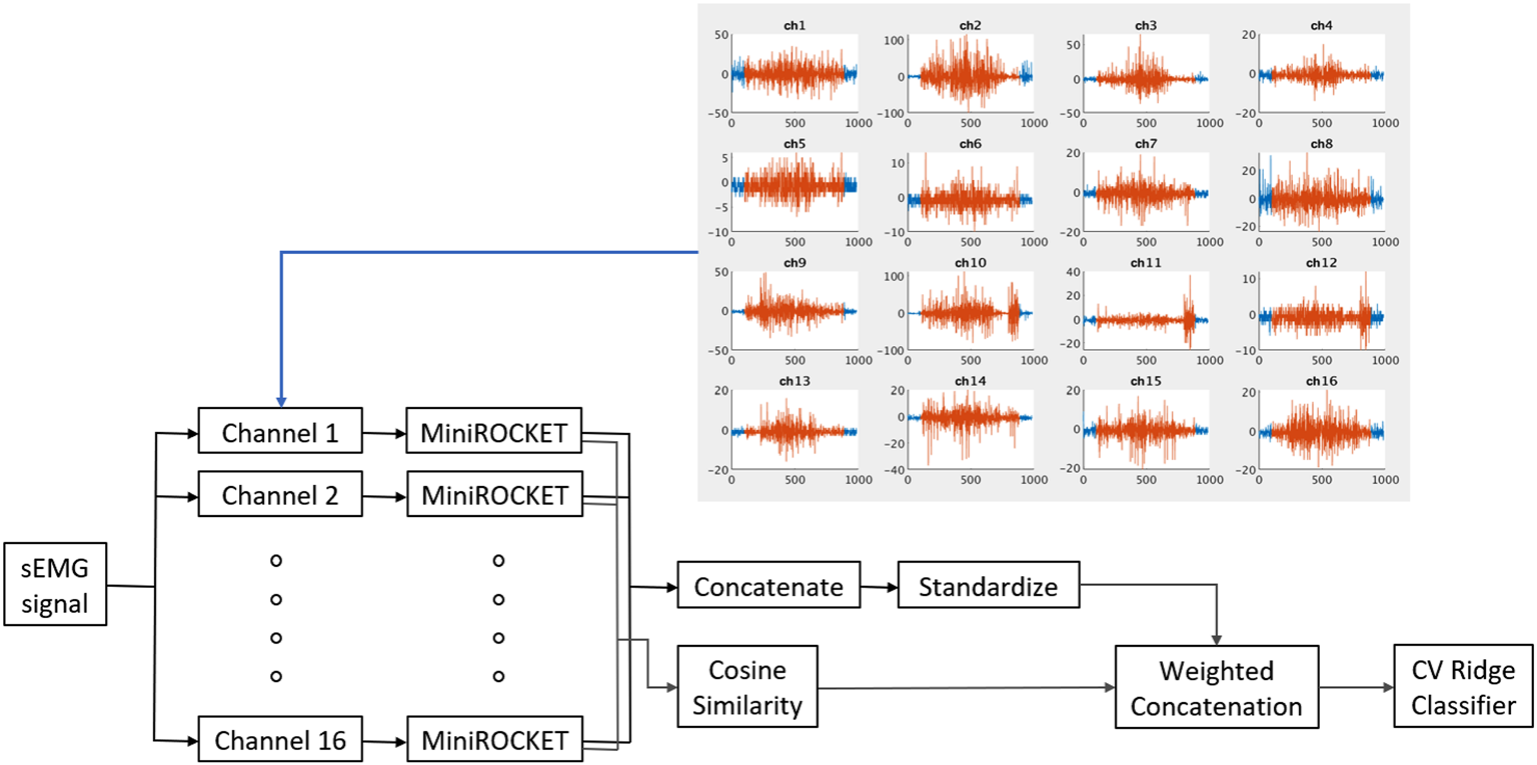
Algorithm flow. MiniROCKET extracts features from the input channels of the dataset. Cosine similarities are computed for each movement based on the features of the input channels. Both representations are fed as input to the ridge regression classifier.

Given a training dataset of *N* sEMG signals, denoted by $\{X^{n}\}_{n=1}^{N}$ each containing data from 8 or 16 channels, MiniROCKET with 10, 000 convolution kernels is applied on $\{X^{n}\}_{n=1}^{N}$ to yield a new representation for each of them. If a single movement is recorded by 16 channels, denoted by $\{X^n_i\}_{i=1}^{16}$ then 16 MiniROCKET feature vectors, denoted by $\{{\tilde{f}}^n_i\}_{i=1}^{16}$ are generated. The outputs of the different channels are then concatenated to a features vector (8 or 16 channels, depending on the experimental configuration), denoted by ${\tilde{f}}^n = \{ {\tilde{f}}^n_1, {\tilde{f}}^n_2, \ldots , {\tilde{f}}^n_16.\}$. Then, each feature vector ${\tilde{f}}^n$ is standardized to have mean 0 and variance 1, to remove any bias towards specific features due to scaling or shifting. In this work, we suggest to add additional features that capture the pair-wise similarity between the channels (electrodes). To do so, the MiniROCKET outputs are used to calculate the cosine similarity between each pair of channels. Given a single signal $X^n$, with, for example 16 channels, the cosine cosine similarity is computed and reshaped to be a row vector of size $1 \times (16 \times 16)$, denoted by ${\hat{f}}^n$, The channels’ similarity outputs and the features vectors are then combined using a weighted concatenation. Thus, the final feature vector that represents a single multi-channel movement $X^n$, is given by $f^n = \omega _1 {\tilde{f}}^n + \omega _2 {\hat{f}}^n$, where $\omega _1, \omega _2 \ge 0$, and $\omega _1 + \omega _2 = 1.$ The weights $\omega _1, \omega _2$ were chosen empirically, optimizing results on the validation data. For classification, cross-validation ridge classifier was applied. Figure 2 displays the general flow of the proposed algorithm.

Algorithms 1 and 2 describes the suggested algorithm for data transformation and classification. For training purposes, Algorithm 1 was applied to each sample of the training and validation data sets, resulting with $X_{\text{ train }}$ and $X_{\text{ val }}$. $X_{\text{ train }}$ and $X_{\text{ train } \text{ labels }}$ were used for the ridge classifier fitting, creating a map between $X_{\text{ train }}$ and $X_{\text{ train } \text{ labels }}$ (Alg. 2, Step 1). Then, the fitted classifier was used to predict $X_{\text{ val }}$ labels (Alg. 2, Step 2). The final model was tested on the test data set. Algorithm 1 was applied on the test data set, resulting with $X_{\text{ test }}$. Then, the fitted classifier was used to predict $X_{\text{ test } \text{ labels }}$ (Alg. 2, Step 2).

**Algorithm 1:**
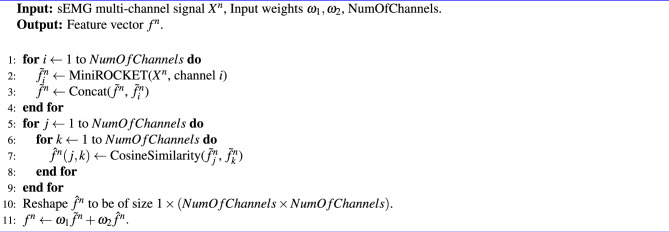
DataTransform.

**Algorithm 2:**
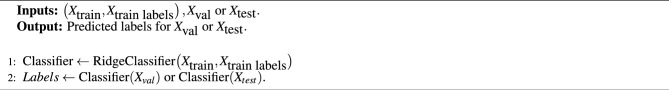
Classification.

## Experimental results

This section presents the results of the suggested ROCKET based classification method.

### Results for the NinaPro datasets

Recall that the NinaPro DB5 dataset includes 6 repetitions per movement (for each subject). Each movement’s repetitions were split randomly to train, validation and test sets, using a 4-1-1 pattern (4 train samples, 1 validation sample, 1 test sample). Additionally, in order to compare with the results of previous studies^4,22^ using the same data set (DB 5 database), an additional non random split was evaluated. Repetitions 1, 3, 4, and 6 were used as training data, while repetitions 2 and 5 were used for validation and test.

**Figure 3:**
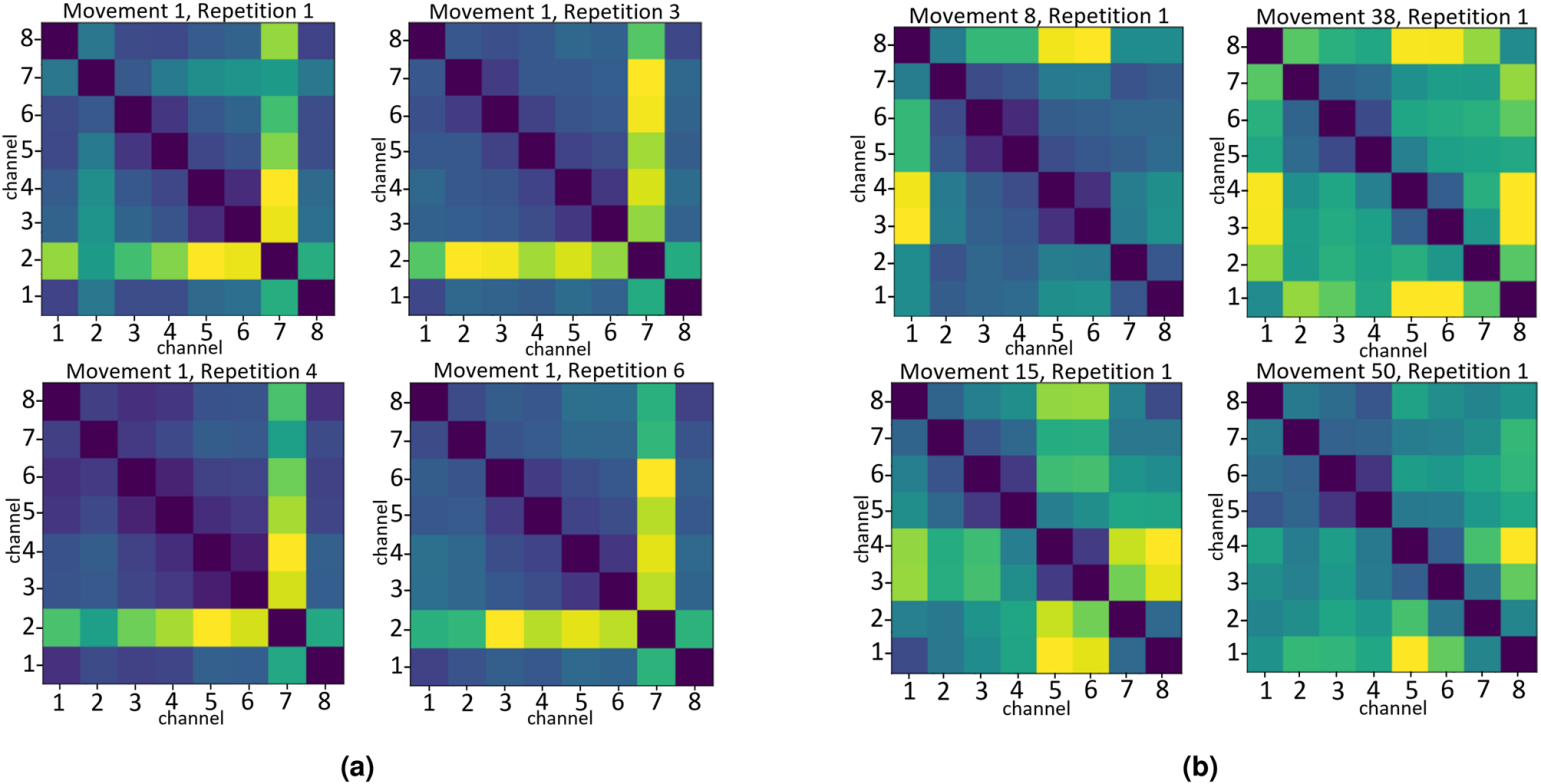
Cosine similarity matrices. (**a**) Cosine Similarity Matrices from the 8 channels data, 4 repetitions of movement 1 (index flexion). (**b**) Cosine Similarity Matrices from the 8 channels data, single repetitions of each of the following movements: 8 (little finger extension), 15 (flexion of ring and little finger), 38 (writing tripod grasp) and 50 (open a bottle).

Figure 3a and b show the cosine similarity matrices for several different movements, using the 8 channels data. Figure 3a shows 4 repetitions of the same movement (movement 1 - index flexion). Figure 3b shows 4 repetition of different movements (movements 8 - little finger extension, 15 - flexion of ring and little finger, 38 - writing tripod grasp, 50 - open a bottle). While the matrices of movement 1 are similar, the other movements differ one from the other. This visual presentation of the cosine similarity data can give motivation for the addition of the cosine similarity to the feature vectors.

As stated in Sect. "ROCKET based classification of sEMG hand and wrist movements", separation between movements and rest periods results with very high accuracy, this result is displayed in Table 1. The first column indicates the number of channels, whether the train-test split was random or not (Y/N). The weight combinations for $(\omega _1, \omega _2)$ are given in the second column. The weights $(\omega _1, \omega _2) = (1,0)$ imply that the cosine distance was not included. It can be seen that in all of the tested configurations rest is easily identified, thus, in the following reported results, rest data was omitted, and the classification results are solely between the movements.

For the classification of 52 movements, the proposed model achieved accuracy (balanced) of $87.69\pm 5.97\%$ for the 8 channels and $94.42\pm 3.59\%$ for 16 channels, using a random 4-1-1 split. When using the non random split mentioned above, the model achieved accuracy of $93.65\pm 2.99\%$ for the 8 channels and $98.27\pm 1.35\%$ for 16 channels. For comparison, previous study^22^ reached accuracy of $93.87\pm 1.49\%$ with a balanced accuracy of $84.00\pm 3.40\%$, while using the non random data split scheme (as mentioned above). Table 2 displays the results of the suggested algorithm while applying MiniROCKET with 10000 convolution kernels. For the experiments that included the cosine similarity matrix, the weights were set to $\omega _1 = 0.3$ for the MiniROCKET features weight, and $\omega _2 = 0.7$ for the cosine distances. These values were chosen empirically, using the validation set, after also considering $(\omega _1, \omega _2) = (0.5, 0.5)$ and $(\omega _1, \omega _2) = (0.7, 0.3)$. The cases for which the cosine similarity isn’t used is equivalent to $(\omega _1, \omega _2) = (1, 0)$.

Additional experiment with a small number of kernels (84 kernels) has been evaluated, results are in Table 3. The number 84 was selected since this is the number of fixed kernels used in the MiniROCKET (see Ref.^19^). In these experiments the cosine similarity matrices were either concatenated with the ROCKET features with the weight combination $(\omega _1, \omega _2) = (0.5, 0.5)$ or not used at all, denoted by $(\omega _1, \omega _2) = (1, 0)$ .

**Table 2:** MiniROCKET classification results (10,000 features).

#	Channels, rand. split	Weights	Validation accuracy	Test accuracy
1	8, Y	(1, 0)	$87.12\pm 5.23\%$	$86.73\pm 6.53\%$
2	8, N	(1, 0)	$85.58\pm 6.04\%$	$92.88\pm 3.22\%$
3	16, Y	(1, 0)	$92.88\pm 2.99\%$	$94.23\pm 3.54\%$
4	16, N	(1, 0)	$93.65\pm 2.99\%$	$98.46\pm 1.68\%$
5	8, Y	(0.3, 0.7)	$87.5\pm 4.96\%$	$87.69\pm 5.97\%$
6	8, N	(0.3, 0.7)	$86.92\pm 5.56\%$	$93.65\pm 2.99\%$
7	16, Y	(0.3, 0.7)	$92.69\pm 3.53\%$	$94.42\pm 3.59\%$
8	16, N	(0.3, 0.7)	$94.81\pm 2.73\%$	$98.27\pm 1.35\%$

**Table 3:** MiniROCKET DB5 classification (84 features).

#	Channels, rand. split	Weights	Validation accuracy	Test accuracy
1	8, Y	(1, 0)	$82.12\pm 5.37\%$	$82.5\pm 5.19\%$
2	8, Y	(0.5, 0.5)	$86.54\pm 4.3\%$	$85.38\pm 5.72\%$
3	8, N	(1, 0)	$82.88\pm 6.05\%$	$89.42\pm 4.06\%$
4	8, N	(0.5, 0.5)	$87.5\pm 3.67\%$	$91.35\pm 4.32\%$
5	16, Y	(1, 0)	$91.35\pm 3.57\%$	$90.96\pm 4.04\%$
6	16, Y	(0.5, 0.5)	$94.62\pm 2.83\%$	$94.81\pm 2.59\%$
7	16, N	(1, 0)	$92.69\pm 4.37\%$	$95.96\pm 2.91\%$
8	16, N	(0.5, 0.5)	$94.81\pm 4.22\%$	$98.27\pm 2.5\%$

**Figure 4:**
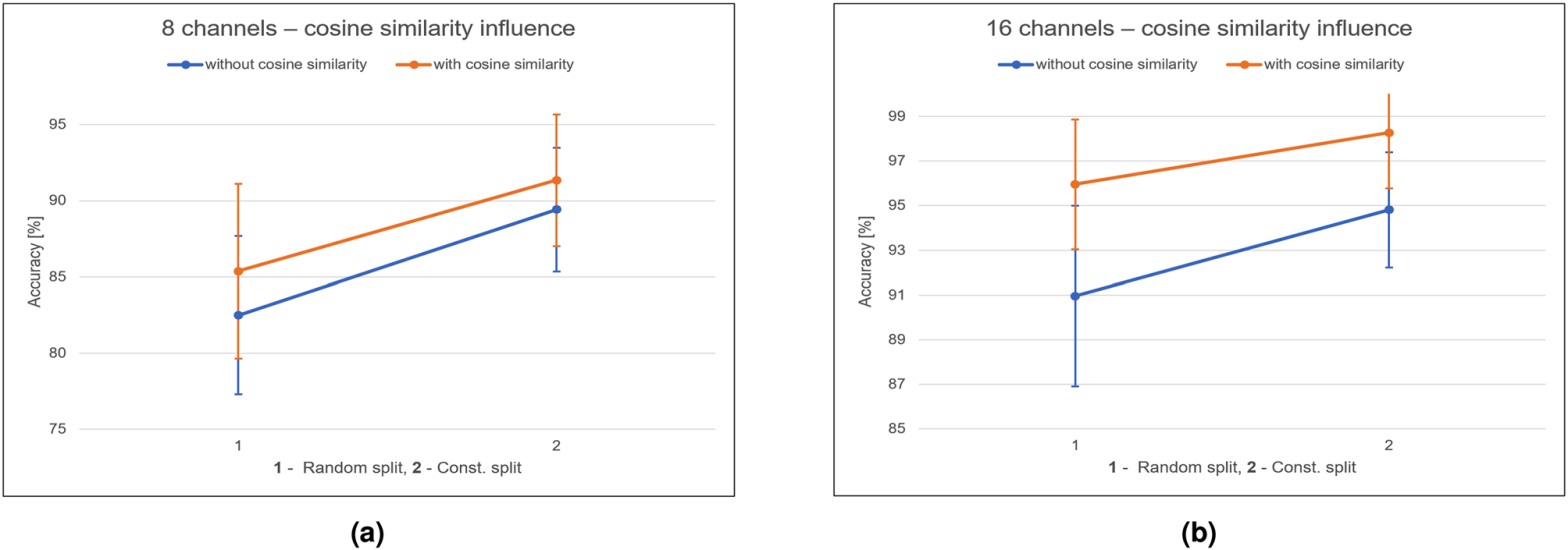
Cosine similarity influence. (**a**) Cosine Similarity Influence, 8 channels data, 84 convolution kernels. (**b**) Cosine Similarity Influence, 16 channels data, 84 convolution kernels.

It can be seen that adding the cosine similarity of the different channels is beneficial to the classification accuracy when less data is available. The 8 channels data set has higher classification accuracy and lower standard deviation relative to the use of MiniROCKET features alone Additionally, when using a smaller amount of kernels (Table 3), the cosine similarity addition has a significant effect on the accuracy, for both 8 and 16 channels (see Fig. 4a,b). Thus, a combination of small amount of kernels and the cosine similarity can keep the accuracy rates high while lowering the overall run time for the feature extraction processing.

**Figure 5:**
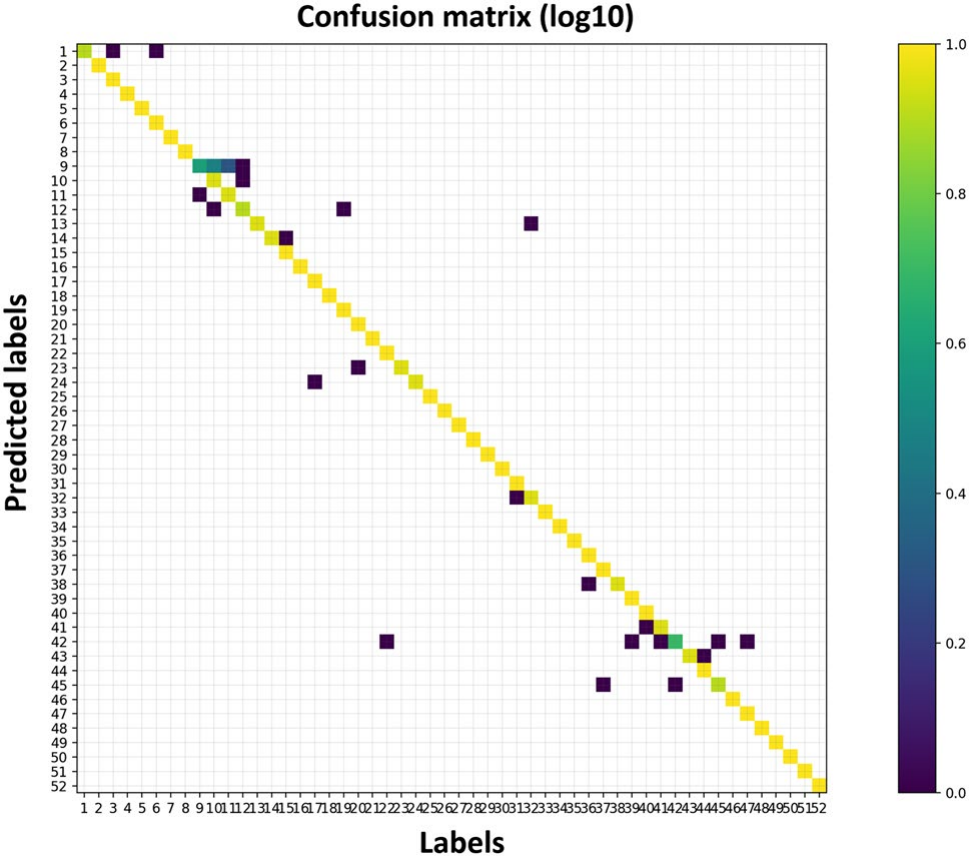
Confusion matrix (log of matrix values), 16 channels data, 84 convolution kernels, Random split, without cosine distance (all missing values were zeros before applying the log function).

It is feasible to pinpoint certain movement groups that the algorithm tends to conflate when making classifications. Figure 5 displays the confusion matrix from the 84 kernels experiment. Movement 9 with movements 10, 11 and 12. All those movement belong to the first category: basic movements of the fingers (Exercise A, see Fig. 1b) all of them are thumb movements. Movement 42 with movements 22, 39, 41, 45 and 47. Almost all those movement belong to the third category: grasping and functional movements (Exercise C, see Fig. 1b). In four of those movement (39, 41, 42 and 45) the grasp movement is similar.

To test the efficiency of MiniROCKET feature extraction, we compared our methods with other known feature extraction methods, described in Ref.^24^. Results are given in Table 4. We used fixed-convolution-based time-domain feature extraction (fcTDFE) and time-domain-based power spectrum descriptors (TDPSD) (see Ref.^23^). Each of the feature extraction methods was applied similarly, and the result were classified using Ridge regression as described in Fig. 2. All algorithms were applied to the DB5 dataset, with 16 channels and a random split.

**Table 4:** Feature extraction comparison.

Method	Weights	Classification accuracy
MiniROCKET (84 features)	(1, 0)	$94.81\pm 3.65\%$
MiniROCKET (84 features)	(0.3, 0.7)	$98.08\pm 1.72\%$
fcTDFE	(1, 0)	$86.54\pm 5.16\%$
fcTDFE	(0.3, 0.7)	$90.77\pm 7.03\%$
TDPSD	(1, 0)	$87.12\pm 5.51\%$
TDPSD	(0.3, 0.7)	$93.85\pm 3.31\%$

**Figure 6:**
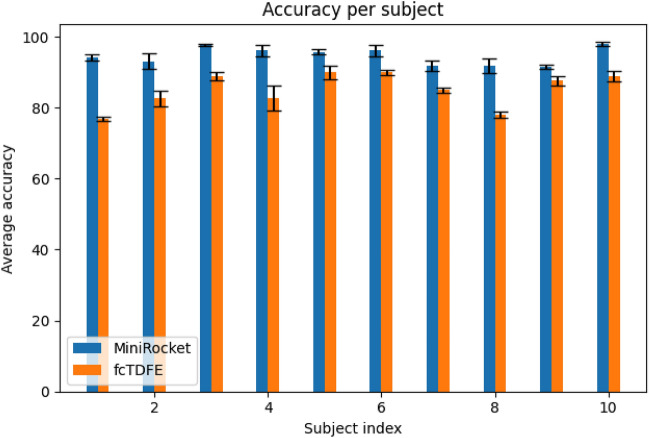
Average accuracy results for MiniROCKET feature extractions vs. fcTDFE feature extraction for DB5.

For statistical analysis we have applied 5-fold cross-validation with $66\%$ of the data for training at each fold, for each of the methods mentioned in Table 4. Results shown in Fig. 6 confirm that MiniROCKET feature extraction provided improved results. Note that *TDPSD* results do not appear in Fig. 6 since they are very similar to *fcTDFE*. To confirm statistical significance of the results, t-tests were applied to each subject. All showed statistical significance (${\text {p-vale}} < 0.01$). As for computational time, a train fold for the ROCKET algorithm takes ${\sim } 30$ seconds, while fcTDFE computation is ${\sim } 21$ seconds. The experiments were conducted on a Macbook pro M1 2020 computer.

In order to test our algorithm on amputees, we used the DB7 dataset, containing 22 subjects, 12 channels and 6 repetitions for each movement. Two of the subjects were amputees. The general results are displayed in Table 5, however it is important to note that the accuracy for amputees only, was at $87\%$. For comparison, in Ref.^22^, reported an overall accuracy of $91.69 \pm 4.68\%$ and a balanced accuracy of $84.66 \pm 4.78\%$. For the two amputees, the results is^22^ were also lower, with overall accuracy of 82.42 and $94.07\%$ and balanced accuracies of 65.10 and $76.55\%$, for the first and second amputee respectively.

**Table 5:** MiniROCKET DB7 classification (84 features).

#	Channels, rand. split	Weights	Validation accuracy	Test accuracy
1	16, Y	(1, 0)	$97.13\pm 3.45\%$	$97.85\pm 5.05\%$
2	16, Y	(0.3, 0.7)	$95.7\pm 4.8\%$	$97.25\pm 5.05\%$
3	16, Y	(0.5, 0.5)	$96.3\pm 4.53\%$	$97.97\pm 3.88\%$

### Results for the UCI dataset

**Figure 7:**
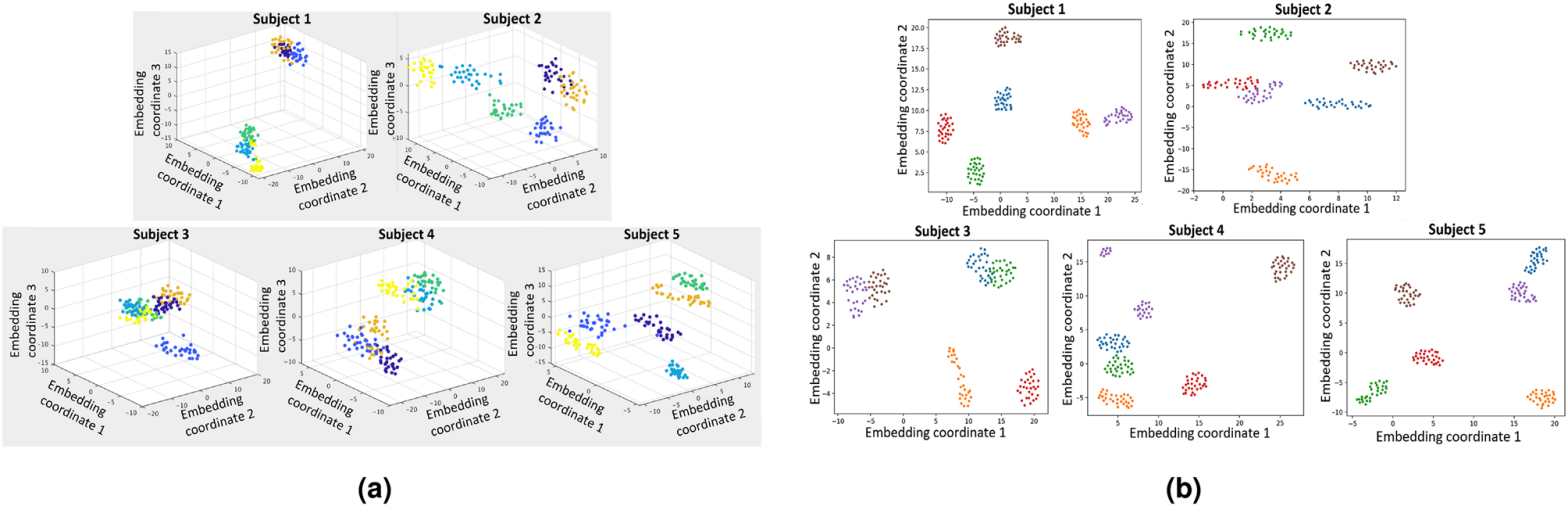
t-SNE and UMAP visualization for STFT features. (**a**) STFT followed by t-SNE, visualization of the separation between the different movements for all five subjects (each movement repetitions appear in different color). (**b**) STFT followed by UMAP, visualization of the separation between the different movements for all five subjects (each movement repetitions appear in different color).

**Figure 8:**
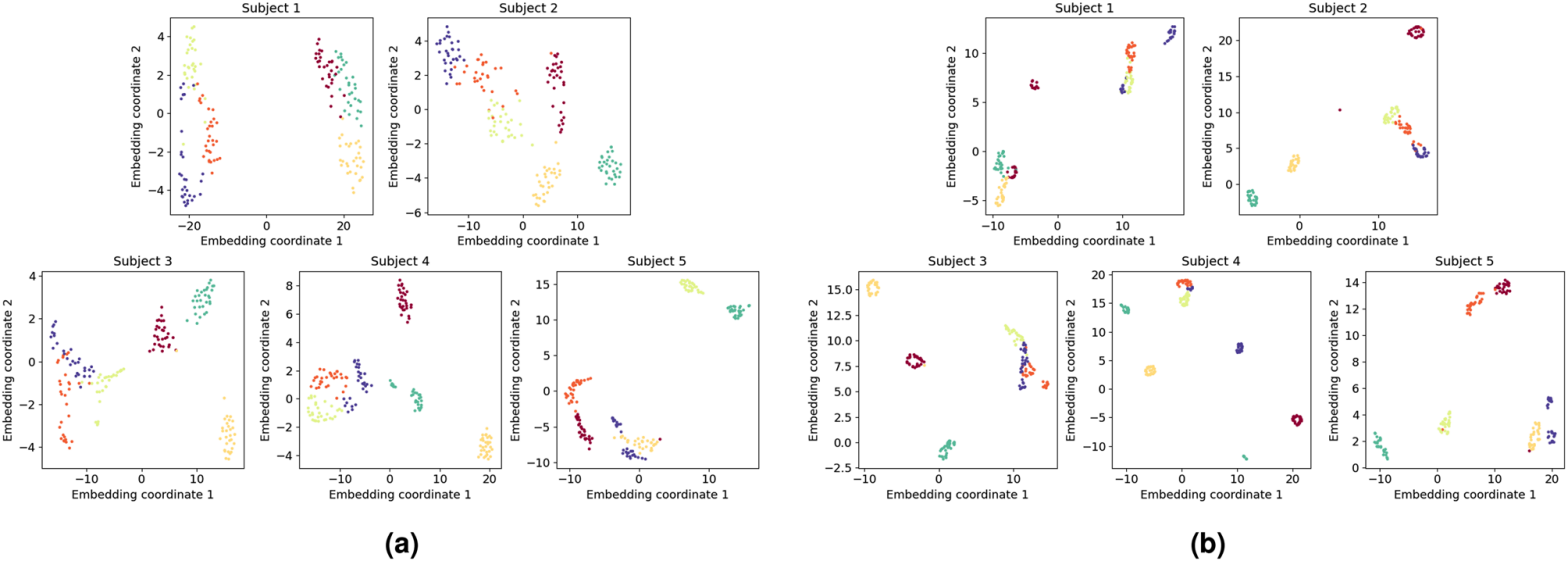
t-SNE and UMAP ROCKET for MiniROCKET features. (**a**) MiniROCKET transform followed by t-SNE, visualization of the separation between the different movements for all five subjects (each movement repetitions appear in different color). (**b**) MiniROCKET transform followed by UMAP, visualization of the separation between the different movements for all five subjects (each movement repetitions appear in different color).

Five different classification methods were tested for the UCI data set. Evaluation was done in a 10-fold cross-validation mode, using $90\%$ of the data for train at each fold. For the first two methods, Short-time Fourier transform (STFT) was applied for features extraction. Next, the STFT train and test images were reduced into a low-dimensional space applying t-SNE^51^ or UMAP^52^. Figure 7a and b show the embeddings for all five subjects. It can be seen that the movements are separated well in the low-dimensional space of both t-SNE and UMAP results. Test points were classified in the low-dimensional space using 3D embedding coordinates and k-NN. For t-SNE, fitting on a training set can’t be used to apply a transform on a test set due to the nature of the method. Hence, embedding was performed on the whole data, kNN was fitted using the training set only and the test set was used for evaluation using the fitted kNN. We also applied t-SNE and UMAP 2*D* classification of the 84 features produced by MiniROCKET (see Fig. 8a,b). Like before, this setting has the limitation that test points were embedded together with the train points, but without their label. Thus, these t-SNE and UMAP models are less applicative for real-time classification. Finally, we test a MiniROCKET configuration with 84 features (standalone and with cosine similarity concatenated), with the ridge regression was used as a classifier. Table 6 summarizes the classification results. It can be seen ROCKET based methods produce the best results, and the proposed method (sketched in Fig. 2) enjoys high accuracy with the benefit of being suited for a real-time setting. The results from the miniROCKET based methods were found to be statistically significant (p-vale $< 0.01$) over the STFT based methods.

**Table 6:** Classification results - UCI dataset.

Method	Weights	Classification accuracy
STFT & t-SNE	(1, 0)	$95.56\pm 5.89\%$
STFT & UMAP	(1, 0)	$89.67\pm 7.37\%$
MiniROCKET and t-SNE	(1, 0)	$99\pm 1.83\%$
MiniROCKET and UMAP	(1, 0)	$98.44\pm 2.54\%$
MiniROCKET and Ridge regression	(1, 0)	$98.44\pm 2.54\%$
MiniROCKET and Ridge regression	(0.5, 0.5)	$98.33\pm 2.54\%$

## Conclusion

This study presents an application of a MiniROCKET based model hand movements based on sEMG. With reported success of the MiniROCKET transform to other time series clasiification tasks^49^, we tested and adapted this robust method and demonstrated the results on public datasets. The public dataset Ninapro DB5 was used as low sampling rate data set recorded using a low cost electrode setup. We tested several configurations of the MiniROCKET features, some combined with a cosine similarity matrix between the channels. As shown in Table 2, when the number of channels is lower (8 instead of 16), the additional channel similarity feature improve the results. Since MiniROCKET computes a large number of feature, we also evaluated the performance degrade when a small number (84 out of 10,000) features are kept. As seen in Table 3, the results are slightly degraded, and the cosine similarity information is needed to maintain high accuracy in this setting. The influence of the cosine similarity matrix for the 8-channel and 16-channel setting was further plotted in Fig. 4a and b. Misclassification was analyzed by using a confusion matrix, displayed in Fig. 5. It indicated that most of the errors occur between movements that belong to the same category of movements, depicted errors happen in the “basic movements of the fingers” and in the “grasping and functional movements” that are presented in see Fig. 1b.

In order to evaluate the strength of the MiniROCKET features, we compared our pipeline, which used the ridge regression as a classifier, with other known feature sets. In particular, we show a comparison with fixed-convolution-based time-domain feature extraction (fcTDFE), proposed in^24^ and time-domain-based power spectrum descriptors (TDPSD) from^23^ in Table 4 and in Fig. 6. The MiniROCKET features show to achieve more accurate classification results.

The same algorithm was tested on the Ninapro DB7 dataset, which includes two amputee subjects. High classification rates are reported in Table 5, and outperform the results reported in^22^, Analyzing the performance of the two amputees alone shows that the results degrade from approximtly $97\%$ to $87\%$, however, these are still much higher than the results that were reported in^22^.

Finaly, for the UCI dataset, the proposed method was compared with a different set of tools that consist of STFT based features combined by dimension reduction. This framework was tested on this dataset in^36^ and achieved a classification accuracy of $94.8\% \pm 3\%.$ Here, similar combinations of STFT with t-SNE and UMAP resulted with slightly higher performance for t-SNE and lower performance for UMAP (as seen in the first two rows of Table 6). Replacing STFT with the MiniROCKET features improved these results (rows 3-4 in Table 6). Nevertheless, the use of non-linear dimension reduction methods isn’t very convenient for out-of-sample extension, thus limiting the use for a real time setting. Therefor, we also tested the two variants of proposed MiniROCKET and ridge regression algorithm, resulting with a classification accuracy that is slightly higher than $98\%$ (last two rows of Table 6), and fits a real-time setting.

For future work, it would be beneficial to test our methods on a larger data set, both subjects and repetitions wise, and on additional datasets that contain recordings from amputees.

## Data Availability

Data supporting the results reported in the article are available from the corresponding author on reasonable request.
